# ERASE-Seq: Leveraging replicate measurements to enhance ultralow frequency variant detection in NGS data

**DOI:** 10.1371/journal.pone.0195272

**Published:** 2018-04-09

**Authors:** Nick Kamps-Hughes, Andrew McUsic, Laurie Kurihara, Timothy T. Harkins, Prithwish Pal, Claire Ray, Cristian Ionescu-Zanetti

**Affiliations:** 1 Fluxion Biosciences Inc., South San Francisco, California, United States of America; 2 Swift Biosciences Inc., Ann Arbor, Michigan, United States of America; 3 Illumina Inc., San Diego, California, United States of America; Mayo Clinic Minnesota, UNITED STATES

## Abstract

The accurate detection of ultralow allele frequency variants in DNA samples is of interest in both research and medical settings, particularly in liquid biopsies where cancer mutational status is monitored from circulating DNA. Next-generation sequencing (NGS) technologies employing molecular barcoding have shown promise but significant sensitivity and specificity improvements are still needed to detect mutations in a majority of patients before the metastatic stage. To address this we present analytical validation data for ERASE-Seq (Elimination of Recurrent Artifacts and Stochastic Errors), a method for accurate and sensitive detection of ultralow frequency DNA variants in NGS data. ERASE-Seq differs from previous methods by creating a robust statistical framework to utilize technical replicates in conjunction with background error modeling, providing a 10 to 100-fold reduction in false positive rates compared to published molecular barcoding methods. ERASE-Seq was tested using spiked human DNA mixtures with clinically realistic DNA input quantities to detect SNVs and indels between 0.05% and 1% allele frequency, the range commonly found in liquid biopsy samples. Variants were detected with greater than 90% sensitivity and a false positive rate below 0.1 calls per 10,000 possible variants. The approach represents a significant performance improvement compared to molecular barcoding methods and does not require changing molecular reagents.

## Introduction

Next-generation sequencing (NGS) has opened the door to personalized medicine by drastically reducing the time and cost required to assess an individual’s nucleic acid composition[1]. This has allowed for the successful identification of germline mutations relevant to inherited genetic disorders[2], cancer predisposition[3], and drug sensitivity[4] among others. It has also provided a means of monitoring tumor material for somatic mutations, providing physicians with crucial information that can guide the course of treatment[5]. The massive read numbers produced by NGS technologies also provide the possibility to look with unprecedented depth into a biological sample, identifying low frequency DNA variants buried at fractions of a percent relative to a wild-type background. One promising application is the liquid biopsy for cancer detection and monitoring which allows for the identification of clinically actionable somatic variants from blood samples, circumventing the need for expensive and invasive repeat tissue biopsies[6–8].

The ability to leverage the high sequence depth provided by NGS instruments to accurately identify ultralow frequency mutations has been technically problematic due to errors introduced by both library preparation and sequencing chemistries[9]. Common bioinformatics workflows utilize metrics such as base quality, read depth, allele frequency, and strand-bias to eliminate the most salient errors in NGS data[10, 11] allowing sensitive and specific variant identification down to 2–5% allele frequency (AF). This has provided a sound methodology for identifying somatic mutations from solid tumor biopsies[5]. However, the cell free DNA obtained in liquid biopsy samples often harbors somatic mutations in the 0.01–1% range[6–8, 12], requiring specialized molecular protocols and algorithms designed to systematically reduce error rates. Molecular barcoding has shown promise in drastically lowering the limit of detection for variants in NGS data. However, the proof of principle experiments demonstrating high accuracy at ultralow allele frequencies have required extremely high read depths and DNA inputs while demonstrating efficacy only over very small genomic regions[13, 14]. Practical attempts to apply this technology to larger clinically relevant sequence spaces have not attained the same performance[15, 16], showing loss of accuracy in the 0.5–2% AF range. A notable exception is the CAPP-Seq iDES method that combines barcoding with a background-aware caller and provides excellent sensitivity to below 0.1%, however high specificity is sacrificed and false positive counts are kept low by confining variant tests to a tiny fraction of the panel[17]. While several cfDNA based tests have been commercialized, significant questions remain as to their fidelity in detecting tumor biopsy variants and agreement across different tests is low [18–20], demonstrating the need for tests with increased sensitivity an ddecreased false positive rates.

**Fig 1:**
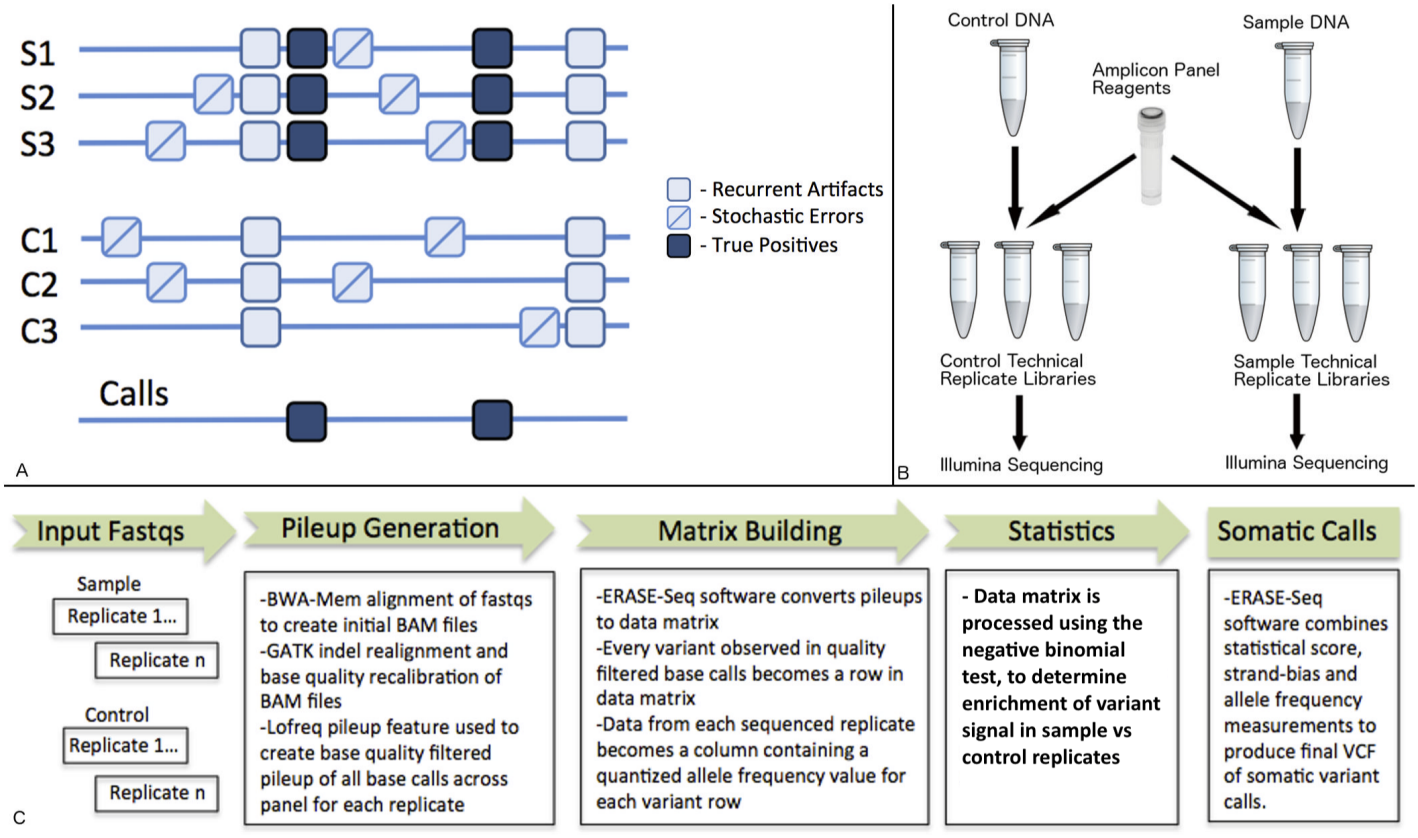
ERASE-Seq concept and method. (A) ERASE-Seq distinguishes true DNA variants from false positives by statistically comparing presence across a series of sample and control technical replicates. False positives arising from recurrent artifacts at error-prone loci (blue squares) are eliminated based on their presence in control replicates. False positives arising from stochastic errors (lined blue squares) are eliminated by inconsistent signal in sample replicates. This allows highly precise detection of true positives (dark blue squares) in final variant calls. (B) The ERASE-Seq molecular workflow is easily applied to amplicon panels by simply preparing and sequencing technical replicates of sample and control DNA in the same fashion they are already being used. Control DNA replicates only need to be generated and sequenced once and can be reused with subsequent samples. (C) The ERASE-Seq bioinformatics workflow begins with BAM file generation and processing of each library replicate. All base calls above a base quality threshold are used to create a pileup for each replicate. ERASE-Seq software converts the replicate pileups to a data matrix representing quantized allele frequencies for each variant in each replicate. The variant data matrix is analyzed using R in order to identify variants that are significantly enriched in sample versus control sequencing runs. These variants are then filtered by strand bias and allele frequency to produce a final set of low frequency somatic variant calls in VCF format.

In the work presented here we apply a new approach to ultralow frequency variant detection. ERASE-Seq combines the power of technical replication with background-aware variant calling (Fig 1) to achieve high-resolution DNA variant calls to below 0.1% allele frequency in highly multiplexed amplicon panels. The basis of our method is elimination of the two major categories of false positives in NGS low frequency variant data: recurrent artifacts and stochastic errors. Recurrent artifacts occur at error-prone loci that are predisposed to base misincorporation either during library prep or sequencing[21, 22]. ERASE-Seq utilizes a set of wild-type control DNA technical replicates that have undergone library prep and sequencing to quantify the error background for each variant across the multiplexed amplicon panel. Stochastic errors also occur during the library preparation and sequencing processes, especially below 0.5% allele frequency. These occur randomly throughout the sequencing space as a function of polymerase error rates[9, 13]. Consequently, they can only be eliminated using technical replication, and we observe decreasing false positive rates with increasing replicate number. ERASE-Seq brings these two error elimination strategies together by employing a statistical test between count values in sample and reference replicates for each observed variant (Fig 1). Recurrent artifacts are eliminated due to their presence in reference replicates, and stochastic errors by their inconsistent signal in sample replicates (Fig 1). We demonstrate the ability of ERASE-Seq to accurately detect low frequency SNVs and indels on three different highly multiplexed oncology amplicon panels: 56G (Swift Biosciences), TST15 (Illumina), and Spotlight 59 (Fluxion Biosciences). Our data show perfect sensitivity and specificity to 0.3% allele frequency and maintain high performance below 0.1%. We analyze the composition of false positive calls using standard methodologies in terms of recurrent versus stochastic errors and show the ability of ERASE-Seq to eliminate these errors. ERASE-Seq represents a significant performance improvement compared to published molecular barcoding strategies sequenced to similar depth and in contrast to molecular barcoding ERASE-Seq can be deployed using existing molecular workflows.

## Materials and methods

### Analytical sample creation

Spiked samples were created by combining DNA from multiple human cell lines. Whole genomic DNA derived from B-lymphocytes was purchased from Coriell Institute corresponding to samples NA12878 and NA19129. Additionally, whole genomic DNA was extracted from cultured tumor cells lines A549, NCI-H1975, and MDA-MB-231 (ATCC) using the Qiagen DNeasy Blood and Tissue Kit (catalog number 69504) followed by qPCR quantification using the ThermoFisher RNase P assay (catalog number 4316831). NA19129 was used as the background sample into which the other four samples were spiked. Two different spike levels were created based on DNA concentrations. A projected 1% spike sample was created such that 96% of the DNA mass corresponded to NA19129 and 1% corresponded to each of the four spiked cell lines (S1 Fig). This sample was then diluted 3:1 with NA19129 such that a projected 99% of the DNA corresponded to NA19129 and 0.25% corresponded to each of the four spiked cell lines. A control sample of pure NA19129 was created in order to quantize the error background for ERASE-Seq variant calling. Samples of pure NA12878, MDA-MB-231, NCI-H1975, and A549 were created to determine true variants present in the cell lines.

### Library preparation and sequencing

Spiked DNA samples and pure NA19129 DNA were used to create targeted amplicon panel libraries followed by sequencing on Illumina machines. The projected 1% spike sample was used as a template for four replicate multiplex PCR reactions using the 56G oncology panel from Swift Biosciences. Pure NA19129 DNA was also used as a template for four replicate multiplex PCR reactions using the 56G panel. Single replicate libraries were made from pure cell line DNA samples NA12878, MDA-MB-231, NCI-H1975, and A549. Manufacturer’s instructions were followed and 10ng of DNA was used as template for each multiplex PCR. These libraries were then sequenced using the MiniSeq 2 x150 high output kit.

The projected 1% spike sample was used as a template for four replicate multiplex PCR reactions using the TST15 oncology panel from Illumina. Pure NA19129 DNA was used as a template for six replicate multiplex PCR reactions using the TST15 panel. Single replicate libraries were made from pure cell line DNA samples NA12878, MDA-MB-231, NCI-H1975, and A549. Manufacturer’s instructions were followed and 10ng of DNA was used as template for each multiplex PCR. The TST15 panel consists of 2 tubes so in total 20ng of DNA was used per replicate. Additionally, the projected 0.25% spike was used as a template for four replicate multiplex PCR reactions using the TST15 panel. Manufacturer’s instructions were followed with two exceptions. First, the DNA input was doubled from 10 to 20ng per multiplex PCR and the number of cycles was reduced from 16 to 15. Second, the number of cycles in the indexing PCR was reduced from 17 to 14. These libraries were then sequenced using the MiSeq 2 x 300 v3 kit, but reads were stopped at cycle 151 on each end.

A second 0.25% spike sample was created and used as a template for four replicate multiplex PCR reactions using the Spotlight 59 oncology panel from Fluxion Biosciences. Pure NA19129 DNA was also used as a template for eight replicate multiplex PCR reactions using the Spotlight 59 panel. Manufacturer’s instructions were followed and 10ng of DNA was used as template for each multiplex PCR. These libraries were then sequenced using the MiSeq 2 x 300 v3 kit, but reads were stopped at cycle 151 on each end.

Raw fastq files for all libraries are available in the NCBI SRA under BioProject Accession Number PRJNA389733.

### Expected variant determination

The analytical performance of ERASE-Seq was tested using standards created by spiking DNA from cell lines A549, NCI-H1975, MDA-MB-231, and NA12878 into a background of NA19129. To determine the expected variants across each panel, libraries were made and sequenced using pure cell line DNA as input. This data was analyzed and germline variants were determined for each cell line across each panel. To determine germline variants from sequencing data, quality and strand-bias filtered pileups were searched for variant alleles at >20% allele frequency. This cut-off provided clear resolution between false positives and true germline variants and allowed identification of germline mutations expected at less that 50% allele frequency due to polyploidies found in cancer cell lines. The expected variant set was then defined as loci variant in one or more of the spiked cell lines, but wild-type in the NA19129 background. These expected variants were used to assess ERASE-Seq sensitivity and specificity and are listed in S3, S4 and S5 Tables. 1 variant was excluded from analysis using the TST15 panel due to poor coverage (7% of mean).

### ERASE-Seq bioinformatics

For the 56G and Spotlight 59 panels primers were trimmed from raw fastq files using Cutadapt[23]. For the TST15 panel primers were trimmed using custom Perl scripts. To test ERASE-Seq performance at lower read depths, down-sampling was performed on fastq files using custom Perl scripts to select a specified number of random reads. We found no performance degradation above a read depth of 5,000x. Trimmed fastq files were aligned to hg19 using BWA-mem[24]. For indel calling, base quality scores were recalibrated and indels realigned using GATK[25]. Lofreq[11] was then used to create pile-ups of all quality-filtered base calls as each position in the panel for all replicates from spike samples and background control samples. Custom Perl scripts were then used to parse pile-up data into a matrix containing read counts for each sample and control replicate at each possible variant. The data matrix was then processed in R using the DESeq[26] negative binomial test to quantify the significance of enrichment between variant count observations in sample and control replicates.

ERASE-Seq exploits the DESeq statistical framework in order to model the probability that a variant exists in sample versus control data. This framework has been demonstrated to provide high resolution in gene expression and copy number studies by accurately integrating replicates from both treated and untreated groups and modeling shot noise present in NGS count data using the negative binomial distribution[26]. A major innovation of ERASE-Seq is to convert replicate variant call data into a matrix than can use this statistical framework, allowing variant calls to be made based on a statistically robust integration of both intrasample technical variation and intersample background artifacts.

ERASE-Seq uses this model to assign a p-value to each possible sample variant call as using the DESeq method previously described[26] For each possible variant if the p-value is above a cutoff/threshold α, then the null hypothesis is true and there is no mutation present in the sample as compared to control runs. Conversely, if the null hypothesis is false, p-value < α, then a mutation call is made for the variant in question. Whereas previously DESeq uses a p-value threshold to determine the presence of a significant copy difference between sample and control runs, here we use a similarly derived p-value threshold to determine the presence of a mutated copy in the starting DNA sample with respect to controls runs.

Subsequently the Benjamini and Hochberg procedure[27] is applied to generate a multiple hypothesis-adjusted p-value to correct for false positives that arise from performing the tens to hundreds of thousands of variant tests across the complete amplicon panel space. The final multiple hypothesis-adjusted p-value adj*P*_*i*_ forms the basis for mutation identification. Custom Perl scripts then integrate adj*P*_*i*_ with strand-bias and allele frequency measurements to produce a final set of variant calls. Allele frequencies for each variant are calculated by taking the mean of allele frequencies observed across all replicates. The strand bias measure used was (AF strand 1/AF strand 2), called Strand Bias Factor. When this factor deviates from 1 in either direction that is indicative of strand bias. In our experiments variants exhibiting a Strand Bias Factor of magnitude > 5 were excluded. Precise adj*P*_*i*_ and allele frequency thresholds used depend on panel and desired limit of detection.

### Sensitivity and specificity calculations

Sensitivity, specificity, and false positive rate are used to compare the performance of ERASE-Seq with several published low frequency DNA variant detection approaches. These metrics are ideal because, unlike other measures like Positive Predictive Value, they are not dependent on the number of true positives in the test sample and thus objectively quantify the performance of the method itself. Sensitivity, or true positive rate, is defined as (True Positives)/(True Positives + False Negatives) and expresses the ratio of known variants in the analytical sample that were called by the method. Specificity is defined as (True Negatives)/(True Negatives + False Positives), and expresses the ratio of alleles known not to be present in the sample that are called as such. In some of the data we chose to instead show false positive rate, which is 1- Specificity. This can also be defined as (False Positives)/(False Positives + True Negatives) and represents the average likelihood that a variant will be called as present in the sample when it is in fact absent. This is alternatively expressed as false positive calls per 10,000 variant tests (False Positive Rate *10,000). These calculations were performed based on our own data and compared to similarly computed metrics from published data to compare the techniques listed in Table 1 and S1 Table.

**Table 1:** ERASE-Seq performance comparisons.

Detection Method	Method Basis	Allele Frequency Range	Sensitivity	False Positives per 10k Variant Tests
ERASE-Seq Spotlight 59	Replicates and background modeling	0.1–0.5%	94%	0.1
ERASE-Seq TST15	Replicates and background modeling	0.07–1.3%	94%	0.7
Integrated Digital Error Suppression [17]	Duplex molecular barcoding and background modeling	0.05–1.6%	97%	<36
Digital Sequencing [12]	Molecular barcoding and background modeling	0.1–1%	66%	not reported
Amplicon Molecular Barcoding [16]	Molecular barcoding only	1–2%	85%	0.2
Molecular Inversion Probes [15]	Molecular barcoding only	0.75–1.5%	72%	0.1

ERASE-Seq is compared to molecular barcoding methods that have been implemented over large target regions and have published performance metrics using analytical standards. Methods that use barcoding alone begin to lose resolution below 1% allele frequency. Barcoding methods that incorporate background modeling can provide sensitivity in the ultralow range but do not demonstrate high specificity. Technical replication allows ERASE-Seq to provide high sensitivity and specificity in the ultralow allele frequency range.

ERASE-Seq, Lofreq2, and some of the molecular barcoding papers analyzed in this study report numbers of false positives across the entire panel. In these cases, the total number of variant tests used for our false positive rate calculations is equal to (Total Number of Base Pairs in Panel)*(3 SNV Tests + 1 Indel Test). This is an approximation because the number of indel tests performed varies by data set. False positive rate metrics are ideal because they characterize the overall number of false positives that will be called per set number of variants considered. If not normalized in this way, the absolute number of false positives will always depend on both the overall panel size and the total number of possible variants considered (variant tests). For example, in the analytical data reported for the iDES [12] method a set of only 279 preselected negative control variants is tested (as opposed to all variants across the entire panel) to determine their specificity, so in our comparison we use 279 as the number of variant tests to determine their false positive rate. Data used to calculate false positive rates and sensitivity for the techniques listed in Table 1 and S1 Table can be found in the following sources: Peng et al: Table 1, Hiatt et al: Fig 4, Newman et al (2016): Fig 4a, Lanman et al: Results; Analytic specificity and sensitivity.

Data for Lofreq2 was obtained by using the same bam files used for ERASE-Seq analysis. These files were recalibrated for base quality and the indels were realigned according to GATK best practices[25]. SNVs and indels were called using default lofreq2 parameters with a homopolymer limit of three. Output vcfs were subsequently tested for sensitivity and false positive rate.

## Results

### Sequencing error composition

**Fig 2:**
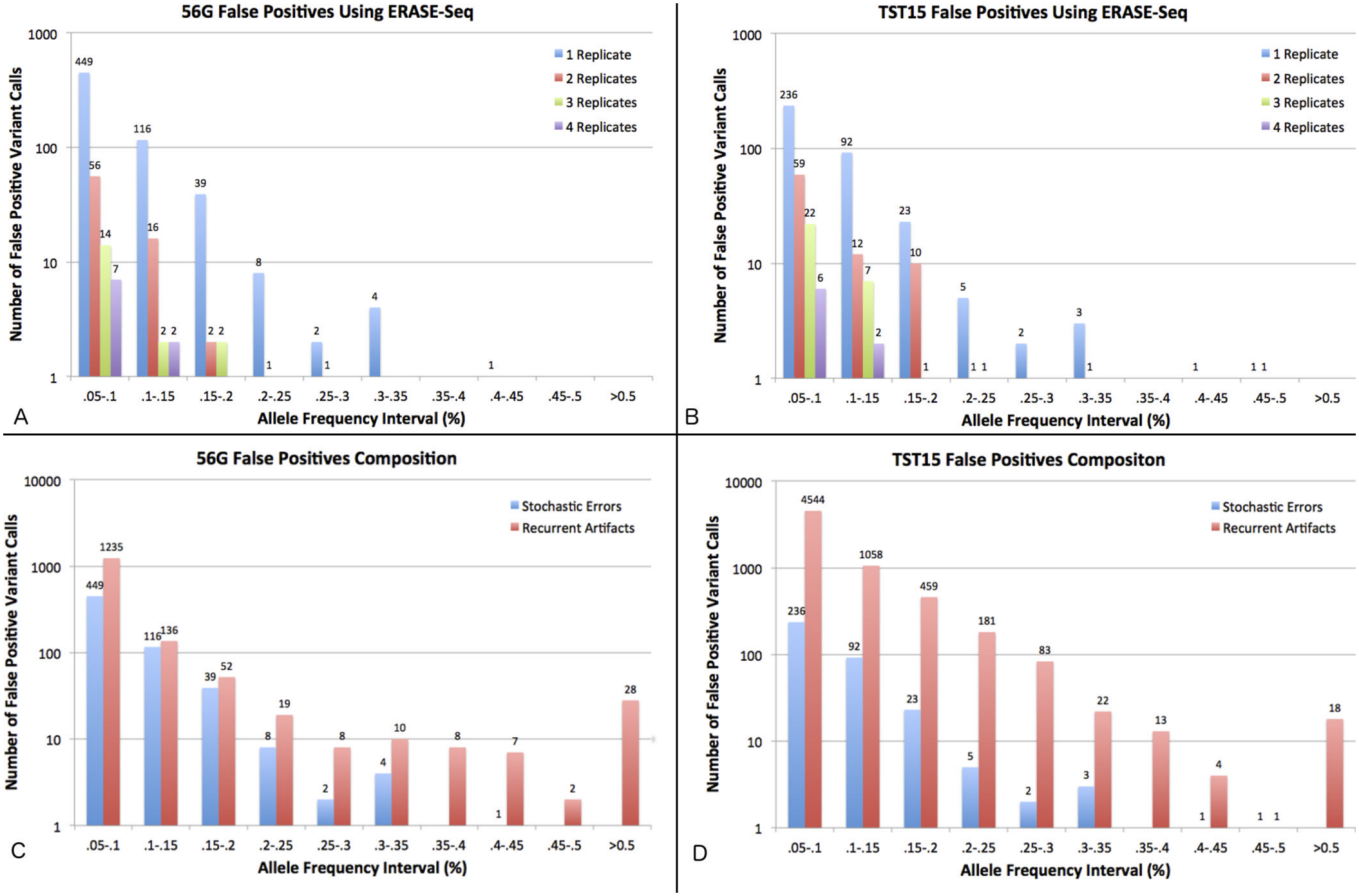
False positive composition. (A,B) The number of false positive calls in 0.05% allele frequency intervals is shown for ERASE-Seq using 1, 2, 3, and 4 replicates for the amplicon panels 56G and TST15. (C,D) The number of false positives using standard intra-sample variant calling metrics (base-quality, strand-bias and read-depth filters) are shown in 0.05% allele frequency intervals for 56G and TST15. They are further divided into recurrent artifacts and stochastic errors. Stochastic errors are those called in single replicate ERASE-Seq and recurrent artifacts are those eliminated in single replicate ERASE-Seq based on the background model.

Replicate experiments against the ERASE-Seq background model allow for an accurate categorization of false positive variant calls observed in sequencing data as either recurrent artifacts or stochastic errors. Fig 2C and 2D shows all false positive calls present in sequencing reads filtered by base quality, read depth, and strand bias for 56G and TST15 experiments. Recurrent artifacts are those false positive calls present in the single replicate data but eliminated by ERASE-Seq due to their presence in the background model. Stochastic errors are those false positives called in single replicate ERASE-Seq, but eliminated with increasing replicate number. False positives are binned by allele frequency and divided into recurrent artifacts and stochastic errors (Fig 2C and 2D). The data show that most false positives are recurrent artifacts at all allele frequencies, with 71% of 56G false positives and 95% of TST15 false positives belonging to this category.

Both types of errors decline in abundance as allele frequency increases although recurrent artifacts are more persistent than stochastic errors. Stochastic errors largely disappear above 0.3% allele frequency and are completely absent above 0.5% in both data sets whereas numerous recurrent artifacts remain in the data above 0.5% allele frequency. Notably, most recurrent artifacts above 0.5% are indels, whereas the majority below 0.5% are SNVs. The general trends observed are the same for both panels but each has its own error profile. TST15 has a 3-4x higher number or recurrent artifacts, but only about ½ of the stochastic errors in AF ranges below 0.2%. This suggests some panel specific optimization of the error suppression protocols is possible. In our experiments detailed below these unique error profiles did not translate into significant differences in ERASE-Seq performance. Spotlight 59 error profiles were not analyzed however the panel chemistry and content is highly overlapping with 56G such that similar results are expected.

### ERASE-Seq eliminates ultralow frequency errors via technical replication

To achieve high specificity at AF < 0.2%, ERASE-Seq uses the statistical power of technical replication. Fig 2A and 2B shows the number of false positives in analytical samples using ERASE-Seq and leveraging 1, 2, 3, and 4 sample technical replicates for both 56G (A) and TST15 (B). False positive counts by bin are shown between 0.05% and 0.5% AF. Robust elimination of false positives at > 0.5% allele frequency occurs due to the background-aware caller, for even a single sample replicate. Below 0.5% the number of false positives increases with decreasing allele frequency, up to hundreds of false positive calls for single replicate samples in the 0.05–0.1% interval. Increasing replicate number maintains high specificity at ultralow allele frequencies. Adding a second sample replicate eliminates 88% of the errors observed with a single replicate for the 56G panel and 77% for the TST15 panel. We have settled on a maximum of 4 replicates, because at 10ng input per replicate this requires a total of 40ng, a quantity obtainable from cfDNA samples isolations. Higher replicate numbers are not feasible, but great results can still be obtained from 2 replicate experiments where input material is limited. Using four replicates allows precise calling down to the lowest allele frequencies. On the TST15 panel only 2 false positives out of 120469 possible variants are called above 0.1% (specificity = 99.9983%) and 8 false positives are called above 0.05% (specificity = 99.9933%). Similarly, on the 56G panel only 2 false positives out of 94888 variant tests are called above 0.1% (specificity = 99.9979%) and 9 false positives are called above 0.05% (specificity = 99.9905%).

### ERASE-Seq performance

**Fig 3:**
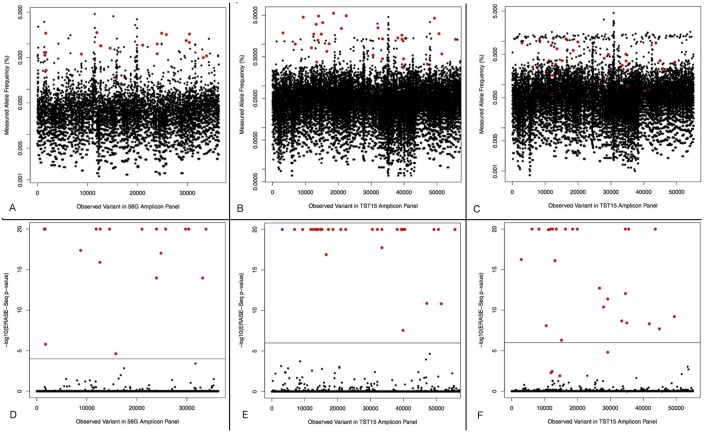
Error reduction using ERASE-Seq. Low frequency variants observed in three analytical DNA spikes mixtures are shown both by allele frequency in the top panel and by ERASE-Seq multiple hypothesis adjusted p-value in the bottom panel. True positives are shown in red and noise is shown in black. (A,D) A spiked DNA mixture is analyzed using the Swift Biosciences 56G amplicon panel. The 19 snvs and one indel ranging from 0.27–1.78% expected allele frequency are detected with perfect sensitivity and specificity using ERASE-Seq. (B,E) A spiked DNA mixture is analyzed using the Illuimina TruSight 15 amplicon panel. The 30 snvs and one indel ranging from 0.35–5.6% expected allele frequency are detected with perfect sensitivity and specificity using ERASE-Seq. (C,F) A more challenging spiked DNA mixture is analyzed using the Illuimina TruSight 15 amplicon panel. The 30 snvs and one indel range from 0.07–1.3% expected allele frequency. All variants above 0.3% allele frequency are detected with perfect sensitivity and specificity and robust detection of ultra-low frequency alleles is achieved with a small number of false positives.

ERASE-Seq eliminated the clear majority of sequencing errors and enabled accurate variant calling to sub 0.1% allele frequency. Fig 3 shows allele frequency measurements and ERASE-Seq p-values plotted for observed variants in three different low frequency spike-in analytical experiments. In the top panel observed variants are shown by allele frequency from a single replicate and true positives are buried among noise from sequencing errors, making accurate calling impossible. The bottom panel shows the same variants’ ERASE-Seq p-value enabling differentiation between true and false positives. The p-value measurement can differentiate all true variants tested from false positives with 100% sensitivity and specificity in the two low frequency experiments (Fig 3A, 3B, 3D and 3E). Ultralow frequency variants shown in the third experiment are still detected with high sensitivity and specificity down to 0.07% allele frequency (Fig 3C and 3F).

### Performance comparisons

ERASE-Seq provides superior variant calling performance compared to previously described low frequency detection methods at similar read depth and DNA inputs (Table 1, S1 Table). Table 1 compares the performance of ERASE-Seq to several other studies that use analytical spike samples to establish performance metrics. In these experiments, DNA with known variants was spiked into wild-type DNA at low relative frequency and libraries made from the spiked sample were sequenced. ERASE-Seq strongly outperforms algorithms based on base quality scores such as Lofreq2[11]. ERASE-Seq is also compared to published strategies using molecular barcoding[15, 16]. Peng et al. and other published methods lose sensitivity and specificity below 1% allele frequency whereas ERASE-Seq maintains perfect sensitivity and specificity to 0.3% allele frequency using both the TST15 and 56G panel. An ultralow frequency analytical spike containing multiple variants below 0.1% allele frequency was also analyzed using the ERASE-Seq. This sample shows 94% total sensitivity and identifies 3/3 variants below 0.1% allele frequency while maintaining a false positive rate of less than 0.007% of variant tests using the TST15 panel. Additionally, an ultralow spike containing variants between 0.1% and 0.5% analyzed using ERASE-Seq with the Spotlight 59 panel demonstrated 94% sensitivity and a false positive rate of less than 0.001%. This demonstrates a large improvement over Guardant digital sequencing which only achieves 66% sensitivity for variants between 0.1–1% allele frequency and does not publish a false positive rate[12]. The method with comparable sensitivity to ERASE-Seq in the ultralow allele frequency range is iDES[17]. However, iDES specificity measurements are taken from an extremely limited search space, testing only 279 preselected negative control variants throughout the panel thus only demonstrating a false positive rate as low as 0.358%. In contrast, ERASE-Seq remains highly specific across the 120,469 variant tests comprising the entire TST15 panel, demonstrating a false positive rate of 0.0064% when calling variants to 0.05% allele frequency. Similarly, ERASE-Seq remains highly specific across the 100,904 variant tests comprising the entire Spotlight 59 panel, demonstrating a false positive rate of 0.00105% when calling variants to 0.1% allele frequency.

### Single replicate ERASE-Seq performance

**Fig 4:**
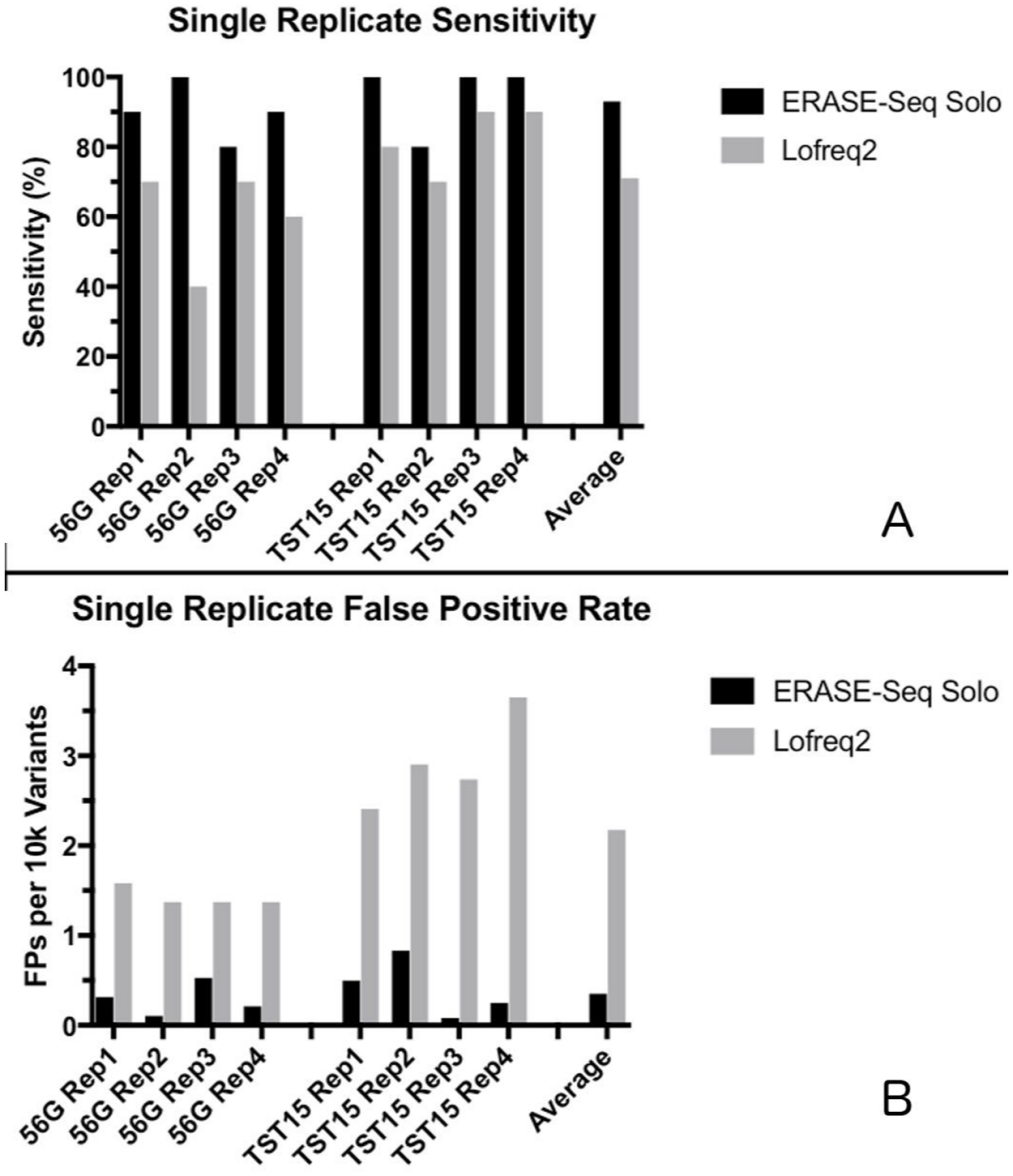
Single replicate ERASE-Seq performance. The ERASE-Seq algorithm may also be used with single replicates to eliminate false positives resulting from recurrent artifacts. This fig demonstrates ERASE Seq’s large gains in resolution below 1% allele frequency as compared to Lofreq2, a high-performing standard low frequency calling algorithm that does not model background errors and therefore does not eliminate recurrent artifacts. Sensitivity in the 0.3–1% allele frequency range is shown along with false positive rate for four analytical samples using the TST15 amplicon panel and four analytical samples using the 56G amplicon panel. ERASE-Seq provides an average increase in sensitivity from 71% to 93% and a greater than six-fold reduction in false positive rate as compared to Lofreq2.

Applying ERASE-Seq to single replicate data provides a background aware variant caller that can be used for typical data generated by targeted sequencing. This analysis demonstrates both the advantages provided by the ERASE-Seq background model and the limitations of variant calling without technical replicates. The same sequencing data used for ERASE-Seq was also analyzed with Lofreq2 (Fig 4). Sensitivity and specificity were measured using the two algorithms across four TST15 samples and four 56G samples, with the same fastq data inputs. The background model-driven ERASE-Seq variant calling algorithm outperforms the base quality score-driven Lofreq2 algorithm. In Fig 4 sensitivity is shown for variants expected between 0.3 and 1% allele frequency, of which each panel contains ten. False positive rates are also shown. ERASE-Seq provides improved sensitivity and reduced false positive rate in all samples analyzed. When compared to Lofreq2, single replicate ERASE-Seq provides an increase in average sensitivity from 71% to 93% and an average 6-fold decrease in the false positive rate (from about 2 down to about 0.3 FPs per 10k variants).

Particularly dramatic gains are achieved for indel calls that are relatively abundant above 0.3% and are recurrent in nature. (Fig 2A and 2B). However, in the space below 0.3% significant stochastic errors exist that cannot be eliminated without replication.

### Practical considerations for ERASE-Seq implementation

Single replicate ERASE-Seq can be applied to existing data sets by creating a background error model to provide excellent performance down to AFs of 0.5%. Its sensitivity is above 90% and specificity is very high, with a false positive rate below 0.3FP/10k variants.

The ideal implementation of ERASE-Seq uses multiple technical replicates sequenced to a read depth of at least 5,000x. The overall depth per sample (at least 20,000x) is not a major challenge, as these depths are commonly achieved in targeted deep sequencing (S1 Table). When using off the shelf reagents, two practical challenges are the additional material costs per targeted amplification reaction and the higher DNA input requirements. These challenges can be addressed by purpose-built kits like Spotlight 59 that provide all the targeted amplification and library preparation reagents necessary for 4 replicates and draw on optimized amplicon chemistries that minimize DNA input and overall reaction tube number. Regardless of the replicate number and amplicon panel chosen, the background needs to only be produced once and can be reused for each sample set.

## Discussion

ERASE-Seq brings together the principles of deconvolution and technical replication to achieve improved performance in ultralow frequency DNA variant calling. The deconvolution component consists of performing repeated library preparation and sequencing of negative control DNA. Each variant in the panel is then quantified across the negative control replicates to establish a variant-specific background noise model. Actual sample measurements are then tested for statistically significant enrichment against the background model to distinguish true positive variants from noise. Previous studies have employed similar deconvolution strategies to increase precision[4, 28] but this is the first background-aware caller to incorporate sample replicates into a robust statistical variant calling model.

ERASE-Seq’s unique approach is to incorporate multiple sample measurements into the statistical test between variant counts in background and sample replicate data. It has been shown that the high degree of noise in NGS data can be mitigated through technical replication and this has been widely adopted in gene-expression studies[29, 30] and suggested for variant calling[9]. ERASE-Seq provides a quantitative statistical model for using technical replicates that is applicable to ultralow frequency somatic variant discovery and provides significant increases in accuracy.

Our data demonstrate the practical power of both the background modeling and replication components of ERASE-Seq. A performance comparison between ERASE-Seq (single replicate) and Lofreq2 applied to the same data set measures the gains of the background-aware caller alone, without use of replicates (Fig 4). Base quality score-driven caller algorithms such as Lofreq2 begin to show false positives below 1% allele frequency[11]. However, the sub 1% variant space is of high interest in clinical liquid biopsy applications[8, 12, 17], so we compare variant calls in the 0.3–1% allele frequency range for single replicate ERASE-Seq. We show improvements in specificity in all eight samples tested while reducing false positive rates by an average of greater than six-fold (Fig 4). These gains are due to the elimination of recurrent artifacts only. Notably, the Lofreq2 caller is equipped with a homopolymer filter that was used in this analysis and was unable to match the power of ERASE-Seq’s empirical background error model for false positive elimination. The variant calling performance metrics are corroborated by our false positive component analysis (Fig 2C and 2D) that demonstrates the clear majority of sequencing errors above 0.3% allele frequency are recurrent artifacts. This also demonstrates the ability of ERASE-Seq to provide large gains to molecular pathology laboratories without altering bench protocols. Laboratories may create and sequence negative control libraries using their current panel reagents. Subsequently sample library preparation and sequencing continue unchanged and resolution can be gained by simply employing the ERASE-Seq variant caller.

Technical replication enables the highest precision in ERASE-Seq experiments. Our false positive component analysis (Fig 2C and 2D) demonstrates stochastic errors begin to accumulate in abundance at ultralow frequencies when using single replicate data. This is because polymerase errors[13, 31] populate the sequencing libraries at ultralow frequencies across the large sequence spaces assayed. These stochastic errors are recalcitrant to elimination because they are present in the sequencing library[9]. Both molecular barcoding and ERASE-Seq technical replication attempt to address this challenge. Replication allows for accurate discernment of the alleles in the biosample whereas base quality-driven algorithms only allow for accurate discernment of alleles present in the sequencing library. The power of technical replication is what gives ERASE-Seq unparalleled specificity in the sub 0.1% allele frequency range, while maintaining high sensitivity. In fact, the sensitivity of the ERASE-Seq method is not limited by inherent background noise, but rather DNA input copy number. For example, 10ng of human DNA input should contain approximately 3333 copies of each allele, meaning that a 0.1% variant will have on average 3 copies. Below this allele frequency variant incorporation in all four replicates becomes inconsistent, following a Poisson distribution. We show that sensitivity in this range can be retained by increasing DNA input to 20ng per replicate by successfully detecting 3/3 sub 0.1% variants on the TST15 panel (S1 Table).

**Fig 5:**
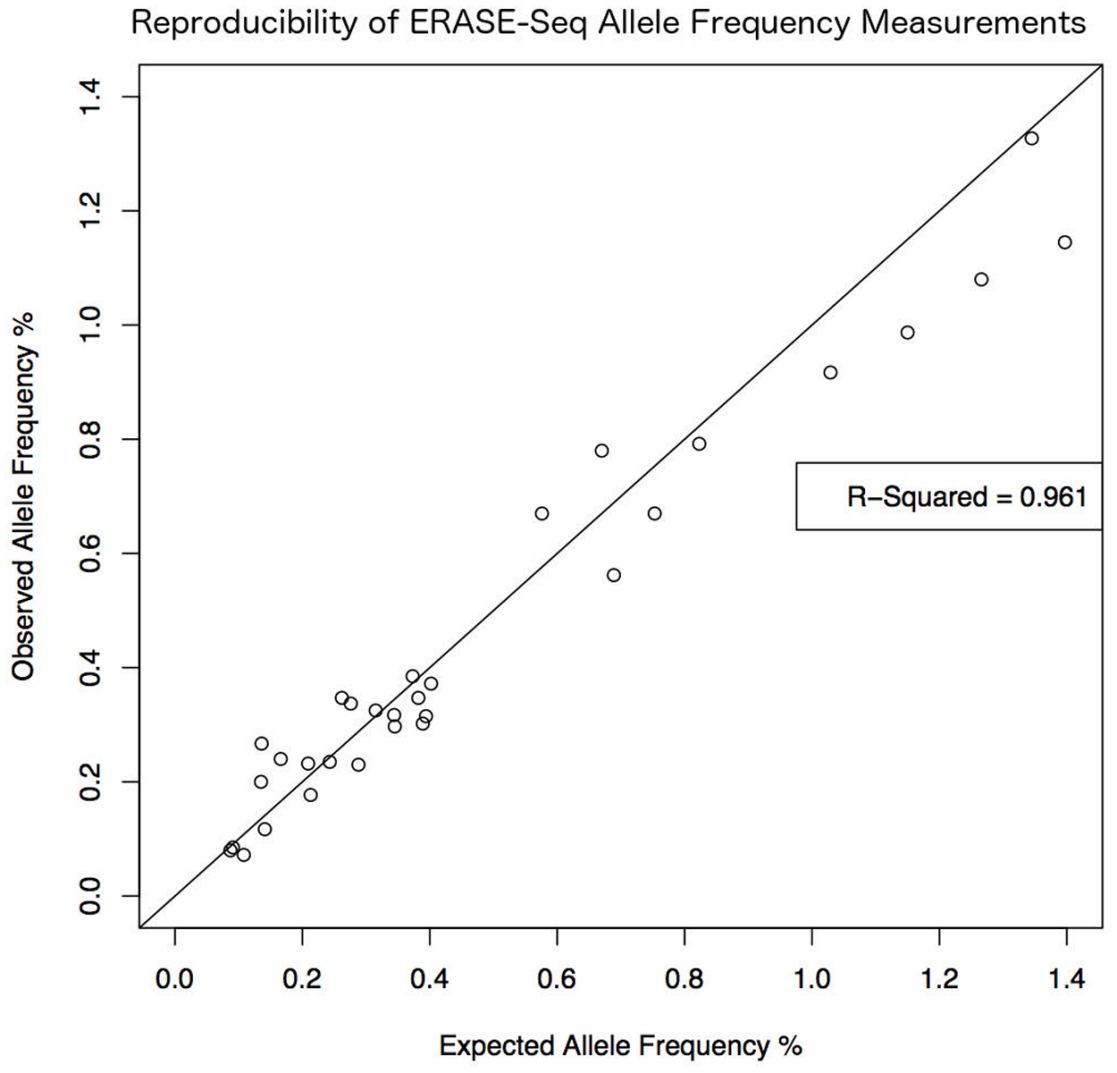
Observed vs expected allele frequencies. ERASE-Seq demonstrates high reproducibility (R-squared = 0.961) in allele frequency determination between experiments, even in the ultralow allele frequency range. This graph compares measured allele frequencies between the 1% TST15 spike and the 0.25% TST15 spike. The 0.25% spike is a simple 4X dilution of the 1% spike into the same NA19129 DNA background so variant allele frequencies in the 0.25% spike are expected to be ¼ their value in the 1% spike. The y-axis plots observed variant allele frequencies in the 0.25% spike and the x-axis plots their expected values.

The wide applicability of ERASE-Seq is demonstrated by successful use of this method with three different oncology amplicon panels and two different DNA sample types. The performance gains observed are consistent across the panels tested showing the robustness of ERASE-Seq. Over 90% sensitivity down to AFs below 0.1% was obtained while maintaining extremely low false positive rates. Perfect sensitivity and specificity is demonstrated down to 0.3% allele frequency. The false positive profiles are highly consistent as is the performance of single replicates. In addition, experiments show high correlation between observed and expected allele frequencies (Fig 5, S2 Table) and training parameters remain robust over repeated analytical tests.

**Fig 6:**
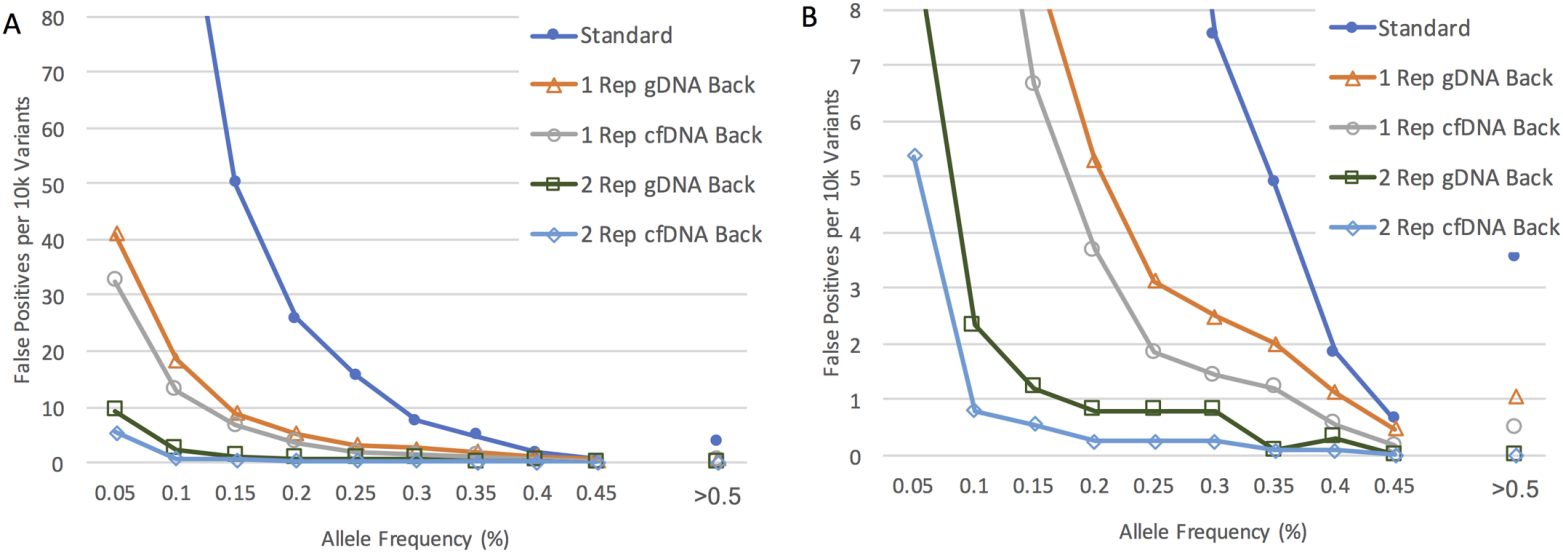
Robustness of the ERASE-Seq approach across different sample types. We analyzed a previously produced data set looking at a Horizon cfDNA standard spike (fragmented DNA) using both an unrelated gDNA background standard and a more similar Horizon cfDNA standard. The false positive rate per 10,000 variant tests is plotted for all conditions. ERASE-Seq results from applying a background model using either background (empty triangle, circle) show a high reduction in the false positive rate for both as compared to a standard caller (filled round). Of the two, using a similar Horizon cfDNA background (empty circles) provides slightly better error correction, while both perform very well above 0.5% allele frequency. The same relationship holds when using two replicates for the Horizon cfDNA sample (square, rhombus), with very low false positive rates above 0.2%. Together, the data demonstrate consistent performance of the background model across sample types. A summary of the false positive rate dependence on the replicate number and control background data used is shown in S6 Table.

A key implementation question is the applicability of error backgrounds obtained using gDNA standards across other sample types. We have addressed this by analyzing an external data set using the Horizon cfDNA standard consisting of fragmented DNA that aims to replicate cfDNA patient samples, and TST15. The data (Fig 6) demonstrates a slight advantage when also using fragmented cfDNA, but the gains are similar in order of magnitude when using our standard gDNA derived background. False positive rates are summarized in S2 Fig and S6 Table. This data set is important in demonstrating the ERASE-Seq approach to be highly efficacious even when different sample types (gDNA vs fragmented DNA) are used to generate the position-specific background model. It demonstrates that the vast majority of errors are introduced during targeted amplification, library preparation and sequencing. The nature of the starting sample only drives a small minority of errors, accounting for less than 5% of false positive calls in our analytical experiment (Fig 6).

ERASE-Seq strongly outperforms molecular barcoding strategies at similar read depths and inputs (Table 1). iDES, a recently developed method that combines a background-aware caller with molecular barcoding, demonstrated great sensitivity in the ultralow frequency range but without the high panel-wide specificity of ERASE-Seq. The iDES approach used a very limited search space (279 variants) to limit false positive calls. This approach to reducing FP rates can be very useful clinically if only a subset of less than a few hundred variants are relevant to disease assessment, but suffers from clear limitations when analyzing wider mutation spectra. Another commonly employed cfDNA assay, the Guardant 360 test also employs a combination of molecular barcoding and background modeling to increase performance[12]. Previously published analytical data for Guardant 360 demonstrate a sensitivity of 66% for variants present between 0.1–1%[12] as compared to over 90% for both iDES and ERASE-Seq[12, 31]. The greatest loss in sensitivity is at the 0.1% level, where only 33% of variants are detected. Analytical specificity of the test is not reported below AF values of 2.5%, nor are the bioinformatics details describing how calls are made[12]. It is likely that search space reduction is also necessary to decrease the false positive rate.

Many recent publications[6–8, 12, 17] report that somatic variants in cancer patient cfDNA are commonly found in the 0.05 to 0.5% range, so increases in sensitivity and specificity will significantly impact the effectiveness of these assays. Even in the advanced patient populations (predominantly stage IV patients), about a third of samples tested do not detect any somatic variants when using the Guardant 360 test[12]. Several recent publications looking and this and other tests challenges in demonstrating high concordance to solid tumors and agreement between different tests[18–20, 32]. This is likely due to a combination of low cfDNA content in these samples and assay sensitivity limitations, so improved sensitivity/specificity will directly result in liquid biopsies being applicable to a larger percentage of patients, earlier in the disease process, with fewer false positives.

Search space reduction can also be combined with ERASE-Seq with the advantage of inherently lower false positive numbers, but we chose not to take this approach. Because the list of clinically relevant mutations is evolving quickly we believe that a whole panel caller is superior. Additionally, there are situations where the high specificity of ERASE-Seq is paramount. Tumor suppressor genes including TP53[33], RB1[34], and Notch1[35] have been shown to have wide loss of function mutation spectra distributed over multiple domains and to include missense, nonsense, and frameshift subtypes. Coupled with the observation that many cancer types can arise from loss of function in one of several tumor suppressor genes[36, 37], the ability to query larger sequence spaces is necessary to avoid false negative clinical results. The analytical work here presents a versatile platform for high-resolution detection of low frequency DNA variants that is particularly suited to the expected allele frequencies found in liquid biopsy samples. Furthermore, ERASE-Seq is the first ultralow allele frequency DNA variant detection method that does not require the adoption of new molecular reagents, making it easily implementable for research and clinical laboratories.

## Conclusions

Liquid biopsy testing methods are being developed at an rapid pace due to the promise of the technology to better represent the diversity of solid tumors and their ability to follow disease progression longitudinally and noninvasively. While a number of recently developed targeted panels and clinical tests have shown great results in selected advanced patient populations, recent literature also highlights significant limitations. Limited sensitivity and specificity have led to measurements of low concordance for CLIA approved cfDNA tests [20] and low sensitivity to solid tumor mutations [32]. Further performance improvements are needed to address a patient populations where at a majority of variants are in the 0.1–1% allele frequency range. Here we present analytical validation data for ERASE-Seq, a new method that, instead of using molecular barcodes, uses technical replicate analysis and a sequencing noise background model to make calls. Using analytical standards on par with previously described methods and clinically realistic DNA input quantities, we detect SNVs and indels between 0.05% and 1% allele frequency with greater than 90% sensitivity and a false positive rate below 0.1 calls per 10,000 possible variants. This provides a 10 to 100-fold reduction in false positive rates compared to published molecular barcoding methods. Future work in patient samples will evaluate clinical improvement by looking at concordance to tumor biopsies and orthogonal high sensitivity PCR-based methods in cfDNA.

## Supporting information

S1 Fig Schematic of analytical sample creation. DNA from four different cell lines was spiked into a normal background (NA19129) at a target of 1% per cell line. Variant allele frequencies present in the analytical mixture spanned a wide range (0.25–5.6%) due to the fact than many variants were shared across some or all cell lines, some variants had non-standard ploidies due to copy number variation, and different quantitation methods led to slightly different input quantities of each cell line relative to the background. This created an analytical sample with many low frequency variants that could be used to test multiple panels. The analytical sample pictured could be further diluted with NA19129 DNA to create samples containing ultralow allele frequency variants.
(TIFF)

S2 Fig Differences in noise between Horizon cfDNA and gDNA samples. The analysis of Horizon cfDNA standards and gDNA allows to compare the noise false positive rate (FP rate) dependence on sample type and DNA fragmentation. Both standard analysis (filled symbols) and ERASE-Seq one replicate only data show a higher noise level for cfDNA as compared to gDNA derived data.
(TIFF)

S1 Table Detailed ERASE-Seq performance comparisons. ERASE-Seq is compared to other low frequency variant detection methods. Performance in terms of sensitivity and specificity is reported in conjunction with sequence depth and DNA input quantity. At comparable sequencing depths and DNA inputs ERASE-Seq outperforms barcoding methods in terms of both sensitivity and specificity. Additionally, ERASE-Seq utilizes moderate increases in sequencing depth to strongly outperform other methods in the ultralow allele frequency range. The * indicates a two-tube multiplexed PCR amplicon panel which is why DNA inputs are higher.
(DOCX)

S2 Table TST15 variant allele frequencies. This table shows the spiked variants along with their allele frequencies across the TST15 panel for the 1% and 0.25% spikes along with the expected allele frequencies of the 0.25% spike. The 0.25% spike is a simple 4X dilution of the 1% spike into the same NA19129 DNA background so variant allele frequencies in the 0.25% spike are expected to be ¼ their value in the 1% spike.
(DOCX)

S3 Table Expected variants in the TST15 panel region. Expected variants in the spiked analytical mixtures are shown along with their allele frequency in each pure spiked cell line. In total 31 variants, 30 SNVs and one insertion, compose the test set. All variants were homozygous for the hg19 reference allele in the pure NA19129 background.
(DOCX)

S4 Table Expected variants in the 56G panel region. Expected variants in the spiked analytical mixtures are shown along with their allele frequency in each pure spiked cell line. In total 20 variants, 19 SNVs and one deletion, compose the test set. All variants were homozygous for the hg19 reference allele in the pure NA19129 background.
(DOCX)

S5 Table Expected variants in the Spotlight 59 panel region. Expected variants in the spiked analytical mixtures are shown along with their allele frequency in each pure spiked cell line. In total 18 variants, 17 SNVs and one deletion, compose the test set. All variants were homozygous for the hg19 reference allele in the pure NA19129 background.
(DOCX)

S6 Table ERASE-Seq efficacy depending on sample type, replicate number and background type. False positives per 10,000 variants tested is shown for ERASE-Seq implementations using a variety of analytical sample types (fragmented cfDNA standard vs gDNA) and background models used. Replicates provide the lowest false positive rates, and function very well independent of sample type.
(DOCX)
