# Supplementary figures and images for: ERASE-Seq: Leveraging replicate measurements to enhance ultralow frequency variant detection in NGS data
Source: PLoS One. 2018 Apr 9;13(4):e0195272. doi: 10.1371/journal.pone.0195272 (PMC5890993; doi:10.1371/journal.pone.0195272)

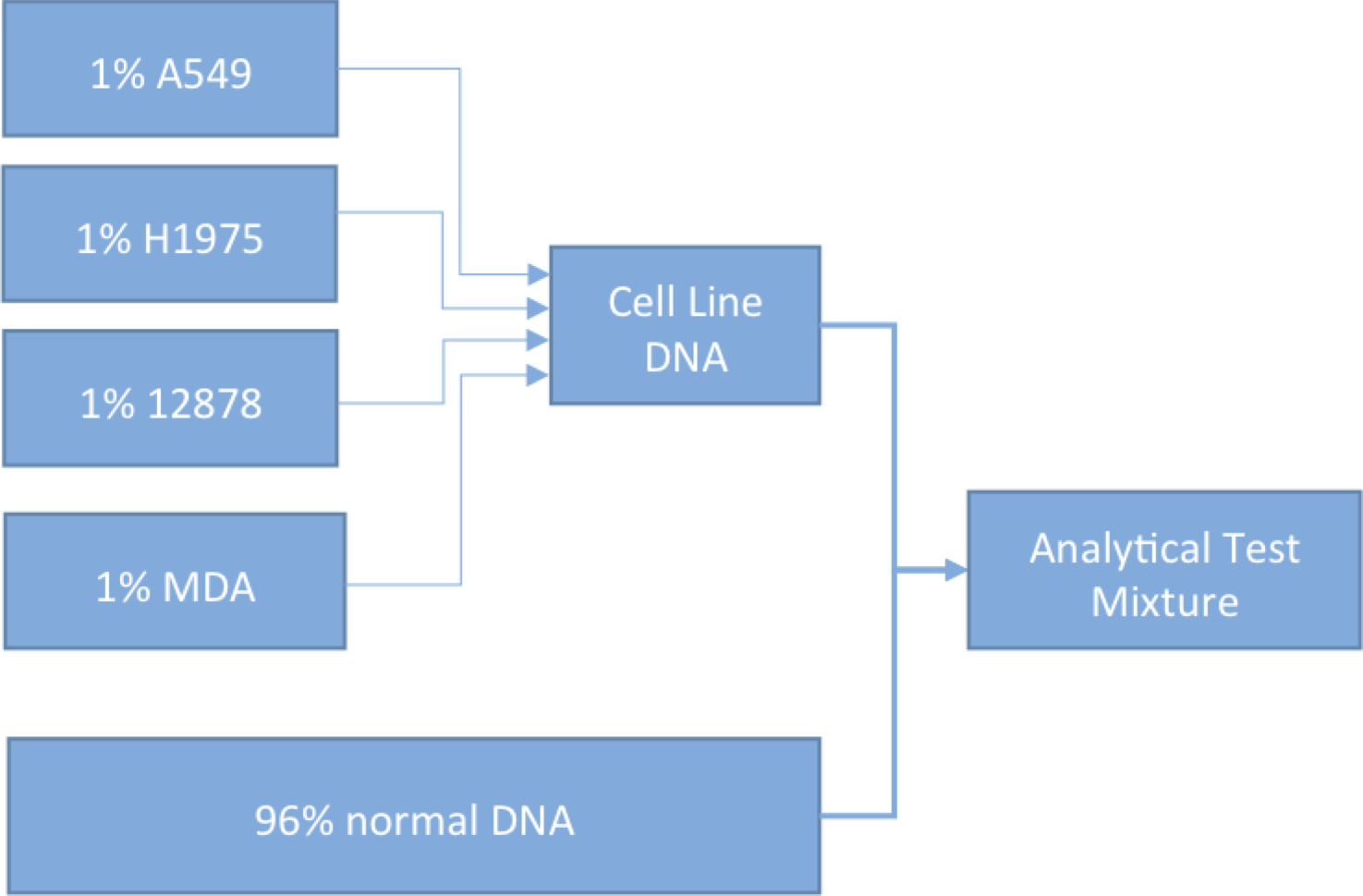

Supplement: S1 Fig — DNA from four different cell lines was spiked into a normal background (NA19129) at a target of 1% per cell line. Variant allele frequencies present in the analytical mixture spanned a wide range (0.25–5.6%) due to the fact than many variants were shared across some or all cell lines, some variants had non-standard ploidies due to copy number variation, and different quantitation methods led to slightly different input quantities of each cell line relative to the background. This created an analytical sample with many low frequency variants that could be used to test multiple panels. The analytical sample pictured could be further diluted with NA19129 DNA to create samples containing ultralow allele frequency variants. (TIFF) [file pone.0195272.s001.tiff]

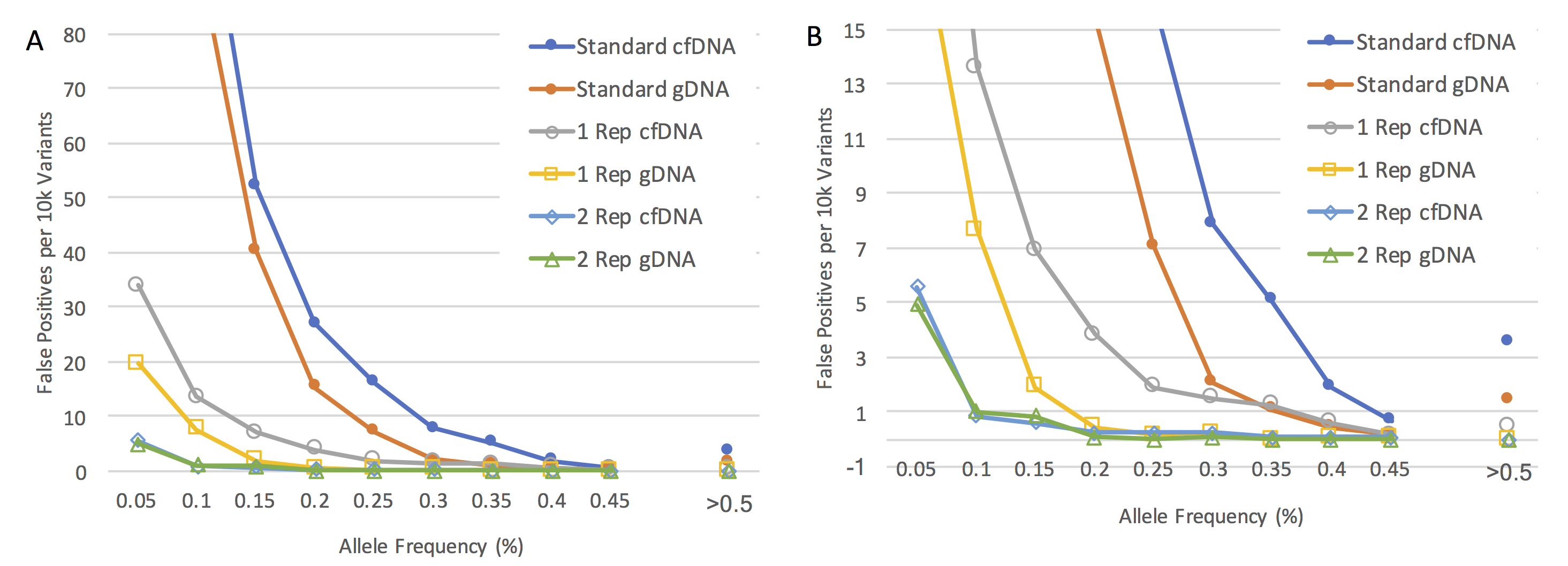

Supplement: S2 Fig — The analysis of Horizon cfDNA standards and gDNA allows to compare the noise false positive rate (FP rate) dependence on sample type and DNA fragmentation. Both standard analysis (filled symbols) and ERASE-Seq one replicate only data show a higher noise level for cfDNA as compared to gDNA derived data. (TIFF) [file pone.0195272.s002.tiff]
