# Supplementary material for: ERASE-Seq: Leveraging replicate measurements to enhance ultralow frequency variant detection in NGS data
Source: PLoS One. 2018 Apr 9;13(4):e0195272. doi: 10.1371/journal.pone.0195272 (PMC5890993; doi:10.1371/journal.pone.0195272)
Supplement: S1 Table — ERASE-Seq is compared to other low frequency variant detection methods. Performance in terms of sensitivity and specificity is reported in conjunction with sequence depth and DNA input quantity. At comparable sequencing depths and DNA inputs ERASE-Seq outperforms barcoding methods in terms of both sensitivity and specificity. Additionally, ERASE-Seq utilizes moderate increases in sequencing depth to strongly outperform other methods in the ultralow allele frequency range. The * indicates a two-tube multiplexed PCR amplicon panel which is why DNA inputs are higher. (DOCX) [file pone.0195272.s003.docx]

| **Detection Method** | **Panel** | **Allele Frequency Range** | **Sensitivity** | **Variants Detected** | **False Positive Rate** | **Median read depth** | **DNA Input (ng)** |
| --- | --- | --- | --- | --- | --- | --- | --- |
| ERASE-Seq 4 Replicates | TST15 | 0.07-1.3% | 94% | 29/31; 3/3 <0.1% | 0.00664% | 52581 | 160* |
| ERASE-Seq 4 Replicates | Spotlight 59 | 0.1-0.5% | 94% | 17/18 | 0.00105% | 40070 | 40 |
| ERASE-Seq 3 Replicates | TST15 | 0.35-5.6% | 100% | 31/31 | <0.00083% | 19451 | 60* |
| ERASE-Seq 3 Replicates | 56G | 0.27-1.78% | 100% | 20/20 | <0.00105% | 20813 | 30 |
| CAPP-Seq iDES^23^ | Capp-Seq NSCLC Selector | 0.05-1.6% | 97% | 28/29; 2/3 <0.1% | <0.35842% | median 14546 | 32 |
| Qiagen Molecular Barcoding^16^ | Custom Amplicon Panel | 1-2% | 85% | 114/134 | 0.00191% | 16275 | 80 |
| Molecular Barcoding^15^ | Custom Inversion Probe Panel | 0.75-1.5% | 72% | 13/18 | 0.00140% | 14364(mean) | 500 |
| Lofreq2^11^ | 56G | 0.27-1.78% | 80% | 16/20 | 0.013700% | 19667 | 10 |
| Lofreq2^11^ | TST15 | 0.07-1.3% | 45% | 14/31 | 0.05479% | 17742 | 20 |
| Guardant Digital Sequencing^12^ | Guardant 360 | 0.25% | 85% | not reported | not reported | 8000 (mean) | 30 |
| Guardant Digital Sequencing^12^ | Guardant 360 | 0.10% | 28% | not reported | not reported | 8000 (mean) | 30 |

S1 Table: Detailed ERASE-Seq Performance Comparison
