# Supplementary material for: ERASE-Seq: Leveraging replicate measurements to enhance ultralow frequency variant detection in NGS data
Source: PLoS One. 2018 Apr 9;13(4):e0195272. doi: 10.1371/journal.pone.0195272 (PMC5890993; doi:10.1371/journal.pone.0195272)
Supplement: S2 Table — This table shows the spiked variants along with their allele frequencies across the TST15 panel for the 1% and 0.25% spikes along with the expected allele frequencies of the 0.25% spike. The 0.25% spike is a simple 4X dilution of the 1% spike into the same NA19129 DNA background so variant allele frequencies in the 0.25% spike are expected to be ¼ their value in the 1% spike. (DOCX) [file pone.0195272.s004.docx]

| hg19 Coordinates | | Alleles | | Variant allele frequencies in analytical spike samples | | |
| --- | --- | --- | --- | --- | --- | --- |
| Chr | Nuc | Reference | Variant | 1% Spike AF | 0.25% Spike Expected AF | 0.25% Spike Observed AF |
| 4 | 55151958 | T | TA | 2.303% | 0.576% | 0.670% |
| 4 | 55602765 | G | C | 0.972% | 0.243% | 0.235% |
| 7 | 55128207 | A | G | 5.065% | 1.266% | 1.080% |
| 7 | 55187715 | G | A | 0.853% | 0.213% | 0.177% |
| 7 | 55214647 | G | T | 1.530% | 0.382% | 0.347% |
| 7 | 55220177 | A | G | 3.291% | 0.823% | 0.792% |
| 7 | 55221167 | A | G | 0.363% | 0.091% | 0.085% |
| 7 | 55222755 | G | A | 1.153% | 0.288% | 0.230% |
| 7 | 55228053 | A | T | 5.382% | 1.345% | 1.327% |
| 7 | 55249071 | C | T | 1.379% | 0.345% | 0.297% |
| 7 | 61088733 | A | T | 3.012% | 0.753% | 0.670% |
| 7 | 63425336 | T | C | 0.838% | 0.209% | 0.232% |
| 7 | 63486408 | C | T | 1.050% | 0.262% | 0.347% |
| 7 | 65425894 | A | G | 5.589% | 1.397% | 1.145% |
| 7 | 65436136 | A | C | 2.757% | 0.689% | 0.562% |
| 7 | 65439879 | G | C | 4.599% | 1.150% | 0.987% |
| 7 | 116398481 | T | G | 0.563% | 0.141% | 0.117% |
| 7 | 116435768 | C | T | 2.678% | 0.670% | 0.780% |
| 12 | 25398281 | C | T | 0.541% | 0.135% | 0.200% |
| 12 | 25398285 | C | T | 1.494% | 0.373% | 0.385% |
| 17 | 7576501 | G | A | 1.578% | 0.394% | 0.315% |
| 17 | 7577099 | C | T | 1.607% | 0.402% | 0.372% |
| 17 | 7577120 | C | T | 1.556% | 0.389% | 0.302% |
| 17 | 7577407 | A | C | 0.434% | 0.108% | 0.072% |
| 17 | 7577427 | G | A | 0.663% | 0.166% | 0.240% |
| 17 | 7577644 | C | G | 1.262% | 0.315% | 0.325% |
| 17 | 25599371 | C | T | 0.346% | 0.087% | 0.080% |
| 17 | 25753033 | T | C | 4.117% | 1.029% | 0.917% |
| 17 | 25901058 | A | G | 1.378% | 0.344% | 0.317% |
| 17 | 25908511 | G | A | 0.544% | 0.136% | 0.267% |
| 17 | 37860994 | A | G | 1.105% | 0.276% | 0.337% |

S2 Table: TST15 Variant Allele Frequencies
