# Supplementary material for: ERASE-Seq: Leveraging replicate measurements to enhance ultralow frequency variant detection in NGS data
Source: PLoS One. 2018 Apr 9;13(4):e0195272. doi: 10.1371/journal.pone.0195272 (PMC5890993; doi:10.1371/journal.pone.0195272)
Supplement: S3 Table — Expected variants in the spiked analytical mixtures are shown along with their allele frequency in each pure spiked cell line. In total 31 variants, 30 SNVs and one insertion, compose the test set. All variants were homozygous for the hg19 reference allele in the pure NA19129 background. (DOCX) [file pone.0195272.s005.docx]

| hg19 Coordinates | | Alleles | | Variant allele frequencies in pure cell line sequencing | | | |
| --- | --- | --- | --- | --- | --- | --- | --- |
| Chr | Nuc | Reference | Variant | A549 | H1975 | MDA | NA12878 |
| 4 | 55151958 | T | TA | 99.82% | 50.28% | 64.82% | 49.38% |
| 4 | 55602765 | G | C |  |  | 35.76% | 47.38% |
| 7 | 55128207 | A | G | 99.79% | 99.88% | 99.79% | 99.65% |
| 7 | 55187715 | G | A | 69.03% |  |  |  |
| 7 | 55214647 | G | T | 30.34% |  | 32.42% | 50.59% |
| 7 | 55220177 | A | G | 33.95% | 99.86% | 34.20% | 50.42% |
| 7 | 55221167 | A | G | 33.54% |  |  |  |
| 7 | 55222755 | G | A |  |  | 33.32% | 54.72% |
| 7 | 55228053 | A | T | 99.72% | 99.69% | 99.84% | 99.72% |
| 7 | 55249071 | C | T |  | 78.82% |  |  |
| 7 | 61088733 | A | T | 32.87% | 79.83% | 99.84% |  |
| 7 | 63425336 | T | C |  |  | 64.00% |  |
| 7 | 63486408 | C | T |  |  |  | 99.81% |
| 7 | 65425894 | A | G | 99.76% | 99.79% | 99.80% | 99.77% |
| 7 | 65436136 | A | C | 99.65% |  | 99.64% |  |
| 7 | 65439879 | G | C | 99.09% | 99.08% | 99.19% | 98.98% |
| 7 | 116398481 | T | G |  |  |  | 46.22% |
| 7 | 116435768 | C | T | 67.03% | 99.94% | 33.83% |  |
| 12 | 25398281 | C | T |  |  | 55.99% |  |
| 12 | 25398285 | C | T | 99.65% |  |  |  |
| 17 | 7576501 | G | A |  |  | 99.84% |  |
| 17 | 7577099 | C | T |  |  | 99.65% |  |
| 17 | 7577120 | C | T |  | 99.75% |  |  |
| 17 | 7577407 | A | C | 21.04% |  |  |  |
| 17 | 7577427 | G | A | 21.21% |  |  |  |
| 17 | 7577644 | C | G |  | 99.53% |  |  |
| 17 | 25599371 | C | T | 22.81% |  |  |  |
| 17 | 25753033 | T | C | 73.94% | 99.50% | 99.50% | 45.73% |
| 17 | 25901058 | A | G |  | 65.63% |  | 50.84% |
| 17 | 25908511 | G | A | 25.69% |  |  |  |
| 17 | 37860994 | A | G | 75.41% |  |  |  |

S3 Table: Expected Variants in the TST15 Panel Region
