# Supplementary material for: ERASE-Seq: Leveraging replicate measurements to enhance ultralow frequency variant detection in NGS data
Source: PLoS One. 2018 Apr 9;13(4):e0195272. doi: 10.1371/journal.pone.0195272 (PMC5890993; doi:10.1371/journal.pone.0195272)
Supplement: S6 Table — False positives per 10,000 variants tested is shown for ERASE-Seq implementations using a variety of analytical sample types (fragmented cfDNA standard vs gDNA) and background models used. Replicates provide the lowest false positive rates, and function very well independent of sample type. (DOCX) [file pone.0195272.s008.docx]

| **Sample** | | **Background** | **Analysis Type** | **FP rate >0.5%** | **FP rate 0.2-0.5%** |
| --- | --- | --- | --- | --- | --- |
| Cell line gDNA Spike | | gDNA Normal Standard | 2 Replicate ERASE-Seq | 0 | 0.3 |
| Horizon cfDNA Spike | | Horizon cfDNA Standard | 2 Replicate ERASE-Seq | 0 | 0.9 |
| Horizon cfDNA Spike | | gDNA Normal Standard | 2 Replicate ERASE-Seq | 0 | 2.9 |
| Cell line gDNA Spike | | gDNA Normal Standard | 1 Replicate ERASE-Seq | 0 | 1.0 |
| Horizon cfDNA Spike | | Horizon cfDNA Standard | 1 Replicate ERASE-Seq | 0.5 | 9.21 |
| Horizon cfDNA Spike | | gDNA Normal Standard | 1 Replicate ERASE-Seq | 1.08 | 15.0 |
| Cell line gDNA Spike | | None | Standard Calling | 1.49 | 26.2 |
| Horizon cfDNA Spike | | None | Standard Calling | 3.65 | 58.3 |
|  |  | |  | *** false positives per 10k variants reported** | |

S6 Table: ERASE-Seq efficacy depending on sample type, replicate number and background type
